# What evidence exists on the interlinkages between ecological and societal impacts of borealisation of the arctic? A systematic map protocol

**DOI:** 10.1186/s13750-025-00367-4

**Published:** 2025-08-02

**Authors:** Kate Baker, Vera Helene Hausner, Jennifer Ramsay, Helen C. Wheeler

**Affiliations:** 1 School of Life Sciences, Anglia Ruskin University, Cambridge, UK; 2 Department of Arctic and Marine Biology, UiT The Arctic University of Norway, Tromsø, Norway

**Keywords:** Climate change, Atlantification, Polar, Communities, Human impacts, Northward expansion, Northward shift, People, Sociological change, Species

## Abstract

**Background:**

As the global climate rapidly warms, one pervasive impact is the “borealisation” of the Arctic. Borealisation occurs when the species, communities and ecological processes of the Arctic transform to resemble that of more boreal lower latitudes. Such change is likely to have profound impacts on the diverse communities and cultures of the Arctic. Some of these impacts are starting to be documented, however this evidence has not been synthesised systematically. This systematic map protocol will therefore address the research question: “*What evidence exists on the interlinkages between ecological and societal impacts of borealisation of the Arctic?”* Additionally, this systematic map will support two current assessments of the Arctic Council working groups on the societal and ecological impacts of climate change in the Arctic, thus responding to policy relevant questions posed by Arctic governments.

**Methods:**

Following guidelines set out by the Collaboration for Environmental Evidence (CEE), a search of literature, both peer reviewed and grey, will be performed using a range of bibliographic databases, websites and search engines. The search strategy will use a pre-defined search string with Boolean operators. The search results will be screened for relevance according to specific inclusion and exclusion criteria. This will be done in two stages – firstly a screen of titles and abstracts, then a full text screening of eligible articles. At both stages, articles will be excluded if they fail to meet all eligibility criteria or if they meet exclusion criteria. Next, articles that are eligible after full text screening will be coded. At both the screening and coding stages, two reviewers will independently assess a defined number of articles to ensure inter-reviewer reliability and resolve differences. This evidence will then form a searchable database with accompanying visual outputs. A narrative output will outline the range and distribution of evidence, identify potential bias, knowledge clusters and gaps, and will explore areas for further research.

**Supplementary Information:**

The online version contains supplementary material available at 10.1186/s13750-025-00367-4.

## Background

As the climate warms, there is a fundamental restructuring of many physical and ecological processes in the Arctic, where the boundary between boreal and Arctic environments is rapidly advancing northwards [1, 2]. This borealisation of the Arctic, as defined in Box 1, is a process whereby the characteristics of the Arctic become more similar to that of lower boreal latitudes. This involves increases in both the presence and density of species more commonly associated with the more southerly boreal region, as well as changes in species traits of Arctic species to resemble those growing and living in boreal areas [3, 4].

The northward movement of species is recognised by the Intergovernmental Panel on Climate Change (IPCC) as a significant global phenomenon in response to climate warming, with very high confidence that half of the species assessed globally have either shifted the distributions polewards or to higher elevations [5].

Borealisation could, as such, result in novel species compositions and interactions triggering a number of ecological and societal impacts. The causes and effects represented are not linear, but interwoven and numerous and are likely amplified by other climate-related impacts that are not directly linked to borealisation.

**Box 1:** Defining borealisation

Borealisation:	
There are different uses of the term “borealisation”. For the purposes of this study, borealisation is defined in terms of ecological changes. Ecological changes are those in the biosphere and have been defined as both “a type of community reorganisation where Arctic specialists are replaced by species with more boreal distributions in response to climatic warming” (3) and “The increased dominance of boreal species traits”(4) as well as increased density of species and northern range expansion of species. The definition of borealisation where human actions have been the primary instigators of a direct change in the Arctic ecosystem will not be used. This definition is illustrated by Roland et al. (2021) where the term “borealisation” was used to describe the active introduction of Norway spruce (*Picea abies*) by humans and consequent replacement of preexisting mixed forests (6). Such direct human interventions will not be included as borealisation in this protocol, as here we are focusing on climate-driven borealisation.	

Across the Arctic, this climate-driven northward expansion of many species is fundamentally transforming ecosystems and this phenomenon is being observed across terrestrial, freshwater and marine systems [6–10]. For example, in terrestrial ecosystems, northward expansion and local increases in height of trees and shrubs adds considerable vertical structure to tundra habitats [11, 12]. Climate-driven borealisation is also proposed to be the main reason for a collapse in the snow crab fisheries in the Bering sea [13]. A heatwave in 2018 and 2019 resulted in a shift from Arctic to sub-Arctic ecosystem conditions, including decline in sea ice, warmer summer bottom temperature likely affecting the metabolism and energy demand of the crab, crab disease, algal blooms, and the increase in crab predators, such as the Pacific cod. Such shifts are usually a result of many interacting drivers that potentially have cascading effects throughout the ecological systems.

**Fig. 1:**
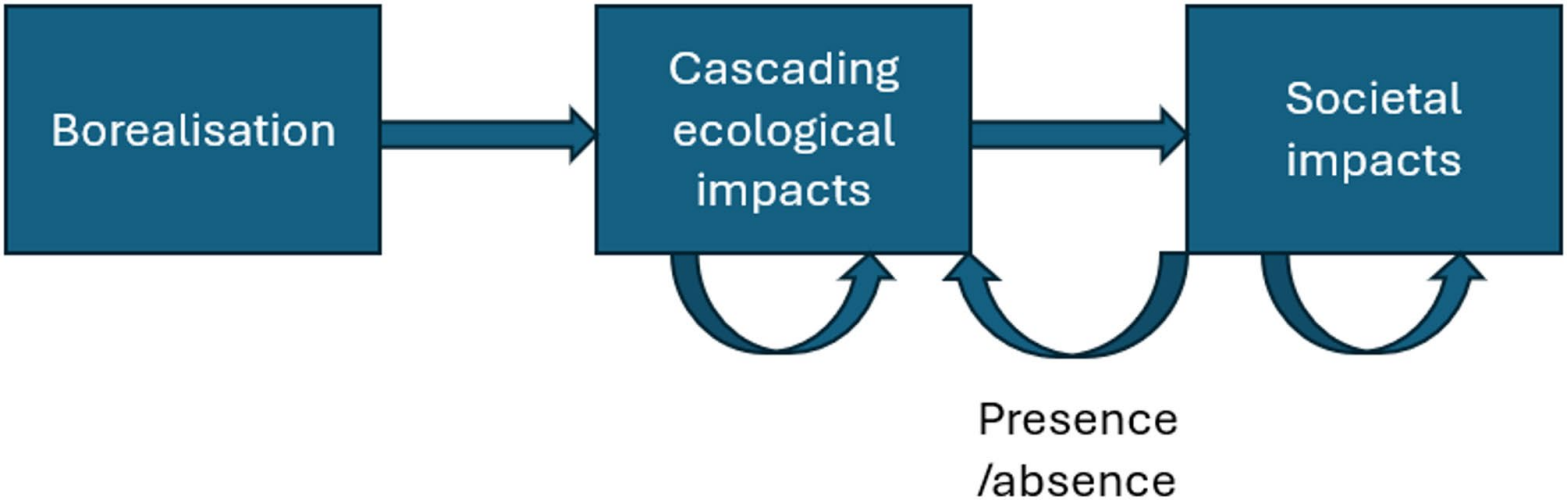
Concept map to show impacts of borealisation on arctic ecosystems and people

While the ecological implications of borealisation of the Arctic are increasingly documented through an expanding literature there are also increasing examples of cascading impacts from climatic change to ecological and societal impacts [14]. These societal impacts of borealisation have received less attention and the existing state of our understanding warrants synthesis. Work is needed to understand and draw linkages between climate-driven ecological change and societal impacts both generally and in the context of borealisation, as well as to identify research gaps which currently limit our understanding of these interlinkages [15] (Fig. 1). This is not only important from a research perspective, but compelling narratives which track the implications of climate change from changes in climate and weather through ecosystem changes to impacts on society are needed to highlight the need for action.

Borealisation can create cascading ecological impacts, these ecological impacts create further feedbacks within ecological systems. These changes within ecological systems can generate societal impacts, which again can create further feedbacks within societies. These will be the primary focus of our systematic map. Societal impacts can in turn feedback to further ecological impacts. We will code only the presence or absence of these feedbacks back from societal impacts to ecological impacts within our map.

Societal impacts can emerge from borealisation via a number of pathways and affect a range of actors. Some impacts relate to holistic responses to change, whereas others result from specific changes in ecosystems. The following examples illustrate some of the impacts that can occur. The transformation of ecosystems can impact sense of place and connection to the land [16]. Changes in the distribution and density of hunted and foraged species may directly affect livelihoods [17], while changes in ecosystem engineers (such as beavers) can cause a series of changes across ecosystems, which can affect travel routes and passage over land and water as well as other species. Differential vulnerability to these changes can emerge from economic, political and societal conditions [18]. Finally, northward expansion of organisms can introduce new diseases to the Arctic [19]. The systematic map will focus on linkages between ecological manifestations of borealisation (in terrestrial, freshwater, coastal and marine ecosystems) and resulting ecological and societal impacts (Fig. 1).

Borealisation is a specific response to climate change that results in a fundamental environmental shift. We choose to focus on borealisation to create a manageable scope that relates to one suite of ecological impacts of climate change and their societal impacts. It also focuses our research on the specific matter of the reorganisation of species and communities as a result of climate change, which is likely to have an impact on societies, while allowing us to address a range of levels of biological organisation from the individual to the population, species and community.

### Stakeholders/Rightsholders engagement

The importance of engaging stakeholders and rightsholders is recognised by the research team. Taking a broad definition of stakeholders, as defined by Haddaway et al. 2017 [20], this includes any groups, individuals or representatives of organisations that could be impacted by or have an impact on the systematic map and its subsequent findings, we also use this definition for rightsholders but add that they also have inherent rights to inclusion related to an issue. Those that we have identified initially, through the research team contacts, are listed below and include representatives from Indigenous Arctic communities, other local communities, research communities, funders and governmental organisations. Other stakeholders and rightsholders may be identified through snowballing of known contacts. It is recognised that biases can occur through these sub-samples and there is a risk of ignoring minorities. However, as the Arctic Council has six permanent partners representing Indigenous Arctic communities, this risk has been partially mitigated. We will engage these groups for their input at two stages - prior to commencing coding where we will ask for feedback on the protocol and while creating presentations and visualisations to ensure the map is usable and relevant for end users. We will prepare summary documents for their feedback on our coding sheet at the first stage and example visualisations at the second stage. We will seek feedback from individuals in the following groups. We will maintain flexibility in our methods for engagement to maximise our capacity to engage and adapt to the specific needs and ways of working of different stakeholders and rightsholder. Our primary mode of communication will be an individual request to provide feedback via email, however we will also create opportunities for individual online meetings and pursue opportunities for virtual and in person presentations at workshops when they arise.

- The Arctic Council (artic-council.org) and relevant working groups and assessment report teams.
  - Conservation of Arctic Flora and Fauna (CAFF): https://www.caff.is/)
  - Arctic Monitoring and Assessment Programme (AMAP):https://climate.amap.no/. Specifically the AMAP & CAFF joint project Understanding Climate Change Impacts on Arctic Ecosystems and Associated Climate Feedbacks: https://www.caff.is/work/projects/understanding-climate-change-impacts-on-arctic-ecosystems-and-associated-climate-feedbacks.
    - /.
- Nodric Borealisation Network (NordBORN): https://www.nordforsk.org/projects/nordic-borealization-network-nordborn.
- International Arctic Science Committee (IASC)and relevant working groups:
  - Marine: https://iasc.info/our-work/working-groups/marine.
  - Terrestrial: https://iasc.info/our-work/working-groups/terrestrial (Terrestrial - International Arctic Science Committee).
  - Social & Human: https://iasc.info/our-work/working-groups/social-human.
- International Arctic Social Science Association (IASSA): https://iassa.org/)

### Objective of the review

The objective of the systematic map is to collate existing evidence on interlinkages between ecological and societal implications of borealisation of the Arctic. We focus on borealisation processes that have impacted people and how they are linked to ecological impacts of borealisation. To do this, we will identfity types of borealisation processes that are occurring in the Arctic, consequent ecological changes and linkages with societal impacts. This will allow identification of knowledge gaps and clusters which could inform priorities for future research and enable understanding of the type and nature of ecological and societal information in papers that assess societal impacts of borealisation.

### Primary question

What evidence exists on the interlinkages between borealisation and societal impacts in the Arctic?

### Secondary questions

- What is the geographic distribution of evidence for societal impacts of borealisation of the Arctic?
- What are the main categories of societal impacts mentioned as a result of borealisation?
- What are the main socio-ecological linkages that have been associated with borealisation?
- What are the disciplinary, methodological and geographic biases in current research on borealisation?
- What are the knowledge gaps and clusters in the research base?

### Components of the questions

- Population: All Arctic habitats(terrestrial, marine, freshwater and coastal) and all human populations across all spatial scales who are impacted by arctic change, both people residing inside and outside the Arctic.
- Exposure: Borealisation of the Arctic (as defined in Box 1).
- Comparator: Before borealisation occurred in the Arctic or using space for time substitution.
- Outcome: Societal impacts on any human communities permanently living in the Arctic, including changes in human activities, health, wellbeing, place-attachment, resilience and vulnerability etc.

## Methods

The methods will follow the *Guidelines and Standards for Evidence Synthesis in Environmental Management* [21] and the ROSES reporting standards for Systematic Map Protocols [22] (See Additional File 4).

### Searching for articles

#### Search language

Due to limited resources and time constraints of the research team, only English language searches will be conducted. The impact of this focus is that it will inform the interpretation of any geographic biases identified by the Systematic Map. While the primary language of peer-reviewed publication in most arctic states is English, it is acknowledged that Russian publications will be under-represented, many of which are in Russian language.

#### Publication year limitations

Due to the limited resources and time constraints of the research team, only articles published after 2009 will be reviewed.

#### Bibliographic sources

Five bibliographic sources will be searched. These have been selected based on their relevance to the field(s) of study and their comprehensiveness.

Searches will be conducted in the following sources:

- Academic OneFile.
- GreenFILE.
- Scopus.
- Web of Science: Core Collection (WoS); all citation indices.
- ProQuest Social Science Database.

### Search string for bibliographic sources

The search string includes a set of each of the following types of terms; Arctic region, borealisation, flora and fauna and societal impact. The string uses the Boolean operators OR to separate terms within a types and AND to separate types. The string is presented below in the Web of Science (WoS) format. The search will be adapted for each bibliographic source to fit their required format. However, the same terms and search fields (Title, Abstract and Key words) will be used.

Anglia Ruskin University (ARU) Library will be used to access content from behind paywalled sources. Where these are unavailable via ARU they will be excluded and recorded as ‘not accessible’.

Search string for bibliographic sources (WoS format):

(Arctic OR Canada OR Greenland OR “north*Finland” OR Iceland OR “north*Norway” OR “north*Sweden” OR Alaska* OR Russia OR Bering* OR Barents* OR “62° to 71°N” OR Polar OR Chukchi OR Siberia* OR “Laptev S*” OR “Kara S*” OR “White Sea” OR “Beaufort*” OR “Norwegian Sea” OR Labrador OR Baffin OR “Davis Strait” OR “Nares Strait” OR “Hudson Bay” OR “Hudson Strait” OR “Wandel Sea” OR “Lincoln S*” OR Lapland OR Lappland OR “MacKenzie River” OR “Ob River” OR “Lena River” OR “Yenise* River” OR Yukon OR “Kolyma River” OR “north slope” OR “Northwest Territories” OR Nunavut OR nunavik OR “nord-de-quebec” OR nome OR “wade hampton” OR bethel OR Dillingham OR Kamchatka OR Chukotka OR Taimyr* OR Taymyr OR Sakha OR Murmansk OR Yamal* OR Nenets* OR Troms* OR Nordland OR Finnmark* OR Norrland OR tundra OR “Attu station” OR “Pruhoe Bay” OR Adak OR Akutan OR Atka OR Fairbanks OR Kenai OR Kodiak OR Kotzebue OR McCharty OR Nikolski OR Seward OR Talkeetna OR Unalaska OR Wasilla OR “Aleutian Islands” OR “Nunivak Island” OR “St Lawrence Island” OR “St Matthew Island” OR “St. Lawrence Island” OR “St. Matthew Island” OR “Brooks Range” OR “Seward Peninsula” OR Yupik OR “Cambridge Bay” OR “Cape Dorset” OR “Clyde River” OR “Coral Harbour” OR “Gjoa Haven” OR “Goose bay” OR “Goose-bay” OR “Haines Junction” OR “Happy Valley” OR “Hay River” OR “Norman Wells” OR “North West River” OR “Rankin Inlet” OR “Repulse Bay” OR “Sawmill Bay” OR “Dawson city” OR Deline OR Igloolik OR Inuvik OR Iqaluit OR Ivujivik OR Kugluktuk OR Kuujjuaq OR Pangnirtung OR Salluit OR Tuktoyaktuk OR Ulukhaktok OR Whitehorse OR Wrigley OR Yellowknife OR “Amund Ringnes Island” OR “Axel Heiberg Island” OR “Banks Island” OR “Bathurst Island” OR “Bylot Island” OR “Devon Island” OR “Ellef Ringnes Island” OR “Ellesmere Island” OR “King William Island” OR “Prince Charles Island” OR “Prince of Wales Island” OR “Prince Patrick Island” OR “Queen Elizabeth Islands” OR “Somerset Island” OR “Southampton Island” OR “Victoria islands” OR “Mackenzie mountains” OR “North west territores” OR “Northwest territores” OR Inuvialuit OR Kitikmeot OR Kivalliq OR Nunatsiavut OR NunatuKavut OR Nunavik OR Nunavut OR Qikiqtaaluk OR Ungava OR “Faroe Islands” OR Faeroe OR Enare OR Ivalo OR Kemi OR Kittila OR Kuusamo OR Rovaniemi OR Torneå OR Ulea OR Uleåborg OR Inari OR “Torne river” OR “north magnetic pole” OR subarctic OR “Søndre Strømsfjord” OR Ilulissat OR Narssarssuaq OR Nuuk OR Sisimiut OR Umanak OR “kalaallit nunaat” OR qaanaaq OR Akureyri OR Isafjordur OR Reykjavik OR “Alta city” OR Bodoe OR Bodø OR Hammerfest OR Harstad OR Honningsvaag OR Honningsvag OR Karasjok OR Kautokeino OR Kirkenes OR Narvik OR Tomsoe OR “Jan Mayen” OR Lofoten OR Varanger OR “Alta river” OR “Pasvik river” OR “Tana River” OR Ivalojoki OR Karasjohka OR “Bristol Bay” OR “Resolute Bay” OR “Alpha ridge” OR “Amerasian bassin” OR “Amundsen Basin” OR “Amundsen Gulf” OR “Cumberland Sound"OR “Eurasian Basin” OR “Foxe Bassin” OR “Fram strait” OR “Gakkel Ridge” OR “Karskoje Sea” OR “Lancaster Sound” OR “Lomonosov Ridge” OR “Makarov bassin” OR “Mendeleev Ridge” OR “Mendeleev Rise” OR “Nansen Basin” OR “Northwest Passage” OR “Okhotsk Sea” OR “Siberian Sea” OR “Viscount Melville Sound” OR Beloye OR Storfjorden OR Anadyr OR Apatity OR Archangelsk OR Chatanga OR Chersky OR Dudinka OR Magadan OR Mirny OR Murmansk OR “Naryan-Mar” OR Nizhnevartovsk OR Norilsk OR Pevek OR Salekhard OR Severodvinsk OR “Tarko-Sale” OR Tiksi OR Urengoy OR Vorkuta OR Yakutsk OR “Franz Josef land” OR “Franz Josefs Land” OR “Novaya Zemlya” OR “Severnaja Zemlya” OR “Wrangel Island” OR Altai OR Anadyr OR Baikal OR “Chamar-Daban” OR Gydan OR Koryak OR Putoran OR Sayan OR “Tannu-Ola” OR Ural OR Verkhoyansk OR Yablonoi OR “Novosibirskije Ostrova” OR Yukaghir OR “Yamal-Nenets” OR Chukotka OR Evenkia OR “Jamalo-Nenetskij” OR Kamchatka OR Karelia OR “Khanty-Mansi” OR Kola OR Komi OR Koryakia OR Krasnoyarsk OR Sakha* OR Yakutia OR Magadan OR Murmansk OR Tunguska OR “Uvs Nuur” OR “Amur” OR “Anabar” OR “Angara” OR “Indigirka” OR “Irtysh” OR “Ob bay” OR “gulf of ob” OR Popigay OR Yana OR Longyearbyen OR.

“Ny Alesund” OR “Ny-Aalesund” OR “Nyalesund” OR “Ny-Alesund” OR “Ny-Ålesund” OR “Bear Island” OR Bjornoya OR Bjørnøya OR Spitsbergen OR Spitzbergen OR Svalbard OR Kongsfjord* OR Hornsund OR Abisko OR Aitik OR Arvidsjaur OR Gállok OR Gárasavvon OR Giron OR Gällivare OR Jokkmokk OR Kallak OR Karesuando OR Kiruna OR Korpilombolo OR Malmberget OR Muddus OR Pajala OR Ritsem OR Soppero OR “Stora sjöfallet” OR Svappavaara OR Vittangi OR Överkalix OR Akka OR Kebnekaise OR Norrbotten OR Padjelanta OR “Sarek” OR Tornedalen OR Tornedalian OR “Vindelfjällens naturreservat” OR “Lainio älv” OR Torneälven OR “Gwich’in” OR “Lower Tanana” OR “Tetlit Zheh” OR Ahtna OR Aklavik)

AND (borealisation OR borealization OR Atlantification OR pacification OR northward* OR “expan* taxa” OR “shifting taxa” OR poleward* OR “ice movement” OR “higher elevation” OR green* OR “climate change” OR “changing climate” OR “environmental change” OR extremes OR warming OR pulse* OR perturbation OR bloom* OR “coastal darkening” OR nitrification).

AND (population* OR species OR communit* OR organisms OR fish* OR game OR herbivore* OR bird* OR mammal* OR plant* OR tree* OR food OR wildlife OR predators OR environment* OR “eco-system” OR “ecoclimatic zone” OR “biome” OR invertebrates OR insects OR arthropods OR disease OR pathogen OR benthic OR pelagic OR reptiles).

AND (societ* OR socia* OR communit* OR human OR “place-based” OR place OR people OR fisher* OR hunt* or fishing OR forag* OR trap* OR harvest* OR herd* OR livelihood OR elder* OR youth OR men OR women OR health OR wellbeing OR well-being OR vulnerability OR resilience).

### Search engine

Google Scholar will be searched to identify any academic or grey literature identified in the bibliographic databases. Using Google Scholar, we will use a simplified search string (below), and only the first 500 results will be downloaded. This approach is based on the limitations of using Google Scholar, as well as the benefits as identified by Haddaway et al. [23] – particularly in identifying organisational reports, government papers and unpublished academic research such as conference papers and theses.

Google Scholar search string:

(Arctic OR Canada OR Greenland OR Finland OR Iceland OR Norway OR Sweden OR Alaska OR Russia OR Bering* OR Barents* OR Polar OR Siberia OR Lapland).

AND (borealisation OR borealization OR Atlantification OR northward* OR “expan* taxa” OR “shifting taxa” OR poleward* OR “ice move*” OR “higher elevation” OR green* OR “climate change” OR “changing climate” OR “environmental change” OR extremes OR warming OR pulse* OR perturbation OR bloom* OR “coastal darkening”).

AND (population* OR species OR communit* OR organisms OR fish* OR game OR herbivore* OR bird* OR mammal* OR plant* OR tree* OR food OR wildlife OR predators OR environment* OR “eco-system” OR “ecoclimatic zone” OR “biome” OR invertebrates OR insects OR arthropods OR disease OR pathogen OR benthic OR pelagic OR reptiles).

AND (societ* OR socia* OR communit* OR human OR “place-based” OR place OR people OR fisher* OR hunt* or fishing OR forag* OR trap* OR harvest* OR herd* OR livelihood OR elder* OR youth OR men OR women OR health OR wellbeing OR well-being OR vulnerability OR resilience).

### Websites

We have currently identified seven organisational websites where this is particularly relevant to complement our Google Scholar search. Websites will be searched to identify relevant grey literature. The searches are all in English so may result in geographical bias in the website searches. This will be reduced by contacting organisations with Arctic area connections who include non-English speaking stakeholders/rightsholders (for example Inuit, Sami, Metis people).

Websites to be searched:

- The Arctic Council (The Arctic Council | Arctic Council (arctic-council.org)) including relevant working group and permanent participant members websites.
- NordBorN (Nordic Borealization Network (NordBorN) | NordForsk).
- Inuit Tapiriit Kanatami (National Representational Organization for Inuit in Canada (itk.ca)).
- Centre for Sami Studies, The Arctic University of Norway.

Welcome to Centre for Sami Studies | UiT.

- Sami University College (Sámi University of Applied Sciences |).
- Reindeer Herding (About - International Centre for Reindeer Husbandry - ICR).
- Inuit Circumpolar Council (Inuit Circumpolar Council – United Voice of the Arctic).

Websites search strings:

*As most websites do not support Boolean operators*,* the following key terms will be used*:

- *Arctic Borealisation and society*.
- *Arctic Borealisation and people*.
- *Arctic Borealisation and Inuit*.
- *Arctic Borealisation and Sami*.
- *Arctic Borealisation and humans*.
- *Arctic Borealisation and community*.
- *Arctic Borealisation and health*.
- *Arctic Borealisation and social*.
- *Arctic Borealisation and societal*.
- *Borealisation*.
- *Atlantification*.
- *People and wildlife change*.
- *People and species change*.
- *Ecosystem change and food*.
- *Ecosystem change and health*.

*We will conduct the website searches using both spellings: borealisation and borealization.*

### Search string scoping

A search string scoping exercise has been conducted in order to test and optimise the search string in both Web of Science and Scopus using Collaboration of Environmental Evidence (CEE) guidelines [21].

Firstly, the number and breadth of articles returned from narrower and wider search strings were tested to obtain a balance between specificity and sensitivity thus reducing the number of irrelevant articles and ensuring all relevant studies were included. This was conducted using 15 benchmark articles (Additional File 1) that were selected based on their relevance to the topic and breadth of studies represented (including borealisation, societal impacts, species changes, trait changes and societal and ecological links). Effort was also made to achieve representation across terrestrial and aquatic examples.

Twenty-four different versions of the search string were trialled. Specificity was assessed and search strings modified according to the number of documents returned by Web of Science in the ‘Topic’ field (including Title, Abstract and Keywords) and Scopus in the ‘Title, Abstract and Keyword’ field. Sensitivity was measured by checking that the key articles had been returned by the search string in Web of Science and/or Scopus.

The search strings selected for use (Additional File 2) were found to be optimum for specificity and sensitivity as they returned all of the key articles and a total of 24,386 reports in Web of Science) and 33,933 in Scopus (search conducted on 12/09/24). The other search strings were discarded for being either too wide (one returning > 15.9 million articles) or too narrow, where not all of the key articles were returned.

### Search update

The search will be updated if original searches are made more than two years prior to the completion of the review. Additional papers will be added to the Systematic Map.

## Article screening and study eligibility criteria

### Screening process

The screening process will be undertaken in two stages:

#### Title and abstract

Records will be assessed for eligibility by title and abstract. Those that meet the eligibility criteria will be included for stage two screening, otherwise they will be excluded. Where there is uncertainty, they will be included in stage two screening.

#### Full text

Remaining articles will be assessed for eligibility based on full text analysis. Those that meet the eligibility criteria will be included, otherwise they will be excluded. Where there is uncertainty, articles will be included and flagged for a second opinion.

The screening process will be conducted using EPPI- Reviewer 6 (EPPI-Reviewer: systematic review software (ioe.ac.uk) [24], a specialist systematic mapping software. Records retrieved from bibliographic sources will be imported to EPPI-Reviewer and all records will be screened after removal of duplicates. Using a set of tick boxes and info boxes, exclusion criteria will be recorded for each article excluded, and a list of reasons for exclusion will be provided for all articles excluded at full text. Those papers that are excluded on the basis that they document only ecological impacts will be flagged and classified to allow assessment of the proportion of borealisation papers that mention societal impacts and for further future analysis of gaps and patterns within a wider set of borealisation papers that exclude societal impacts. Using EPPI-Reviewer, duplicate articles returned by more than one source will be removed.

### Inter-reviewer reliability

To ensure reliability of the screening process and consistency of decisions made, a selection of the records will be screened by two reviewers (double screening). At the first screening stage 100 records will be selected randomly for double screening. A Cohen’s kappa test [25] will be undertaken to check the conformity between both reviewers (inter-rater reliability [26]). Kappa results over 0.6 will be deemed an appropriate level of agreement – any disparity will be discussed and resolved. For kappa scores of less than 0.6, the research team will discuss the eligibility and exclusion criteria and amend where necessary to improve consistency between reviewers. This process will be repeated until the kappa scores are over 0.6 at the screening stage.

At the coding stage, fifteen of the articles will be coded by two reviewers. Any discrepancies between reviewers will be discussed and coding guidelines refined as required to clarify coding decisions. All authors involved in screening or coding will take part in these inter-reviewer reliability processes. Should a member of the review team be listed as an author on any article that appears in the screening process, they will not be included in decisions made about that article. This will ensure procedural independence.

### Eligibility criteria

The following Population, Exposure, Comparator and Outcome (PECO) criteria will be used to exclude or include all articles:

#### Population

All Arctic habitats and human populations that are impacted by arctic borealisation.

- All Arctic habitats will be included – including terrestrial, freshwater, marine and coastal.
- Studies involving impacts on any human populations will be included provided they are impacted by Arctic borealisation. These populations could be inside or outside the Arctic.
- To produce a broad and relevant systematic map, research conducted in all geographical locations of the Arctic as defined by the UArctic taskforce [27] will be included.
- Articles that study multiple regions which include the Arctic will be included.
- Articles that do not study the Arctic region will be excluded.

#### Exposure

Borealisation of the Arctic (as defined in Box 1).

- Studies that involve climate change (or related terms) and evaluate the impact(s) of what is considered borealisation without specifically using the term borealisation will be included.
- All taxonomic groups will be included to provide an overview of evidence of all groups that have been studied. The coding process will allow the evidence for taxonomic groups to be differentiated.
- Studies that address multiple exposures will be included.
- Studies that involve only climatic warming or other physical drivers without reference to ecological groups will be excluded and criteria for exclusion recorded.

#### Comparator

Before borealisation occurred.

- We will include all studies where the comparator is a change in the presence, density or traits of any given species.
- Space for time substitutions intended to examine borealisation will be included.

#### Outcome

- All types of societal impacts will be included e.g. health, wellbeing, place- attachment, resilience and vulnerability and all social and cultural dimensions.
- Studies that address multiple outcomes will be included.
- Studies will be excluded if they only include ecological impacts of borealisation without including implications for society. For comparison of the number of purely ecological studies with those that show societal impacts, we will code these studies to a specific exclusion criterion.
- The search string is likely to return studies involving the impacts that human activities have on ecological aspects of the Arctic (including commercial activities and anthropogenic climate change). As the systematic map relates to the impacts that borealisation of the Arctic has on people (not vice-versa), these studies will be excluded if they do not include societal impacts of borealisation.
- Studies that include ecological-societal feedback loops from borealisation will be included (for example, where the ecological impacts of borealisation impact human societies in ways that lead to humans impacting back on ecological processes). As the detailed coding of outcomes will be specific to societal impacts, any feedback impacts of human societies on ecological processes will simply be coded as present or absent.
- Studies where the implications for society are ambiguous or not explicitly analysed but may still be relevant will be included and coded separately as their documentation within the database could prove useful for future research and analysis of more speculative perspectives on the likely societal impacts of borealisation.

#### Study type eligibility

- Studies that involve simulation or modelling of impacts of borealisation will be included.
- Studies that involve only palaeoecology without offering a modelling element for current and future scenarios will be excluded.
- Review papers and data syntheses will only be included if they offer new insights on the impacts of borealisation on society. This may be in the form of speculation, interpretation or perspectives and the nature of these insights will be coded as speculative or interpretative.

Review papers that do not offer additional insights will be excluded to avoid duplication with original papers that are already included in the systematic map.

We are aware that studies involving the social and ecological sciences will often prioritise one discipline such that information may be richer, more rigorous or deeper from one discipline than another. However, to engage with interlinkages between the ecological and social we need to ensure all information is coded. We therefore intend to use a wide range of evidence types in our systematic map (including articulations of concern of societal impact and speculative propositions). This will allow us to identify emerging themes that warrant more comprehensive study. We hope this will also help us provide guidance to interdisciplinary studies on how to improve the reporting of cross disciplinary evidence to provide clearer and more precise understandings of interlinkages between ecological and societal impacts. Accordingly, all study types that include original data will be included (e.g. experimental and observational field studies, modelling studies, and societal research with interviews, surveys or participant observation) and study types such as reviews and syntheses which add new information through expressed concerns and more speculative data will also be included.

### Study validity assessment

Studies will not be critically appraised as the systematic map will provide an extensive overview of research rather than in-depth evaluation of study quality. Study type and duration will be coded to help with study validity assessment in any future systematic reviews.

### Data coding

For all eligible studies that pass the second screening, meta-data and relevant variables will be extracted and coded as per the coding sheet (Additional File 3) using EPPI-Reviewer 6 software. The coding sheet was adapted from Sechaud et al. (2022) [28].

The coding sheet will be tested by two independent reviewers, using a sub-set of fifteen full-text articles each. To assess inter-reviewer reliability, these articles will be the same for each reviewer. Any inconsistencies will then be discussed and coding guidelines and variables amended where necessary. Further rounds of 15 articles will be double-coded until consistency is achieved. This will allow an assessment of the suitability and comprehensiveness of coding variables and repeatability of the process.

Coding will be conducted through a combination of deductive coding (using a pre-defined set of coding variables) and inductive coding (adding new codes as they emerge from the literature). In articles containing multiple original evidence points (e.g. research questions, studies or data sets), each evidence point will have a unique identification number (ID) and be recorded separately. Where an article contains unclear or missing data for coding variables, corresponding authors will be contacted by email in the first instance. If the data is unavailable it will be recorded as unavailable.

### Study mapping and presentation

The outputs of the systematic map will include a searchable database, visualisations and narrative synthesis. These will describe and map the existing evidence base for societal impacts of Arctic borealisation. Knowledge clusters and gaps will be identified and visualised in relation to the specific review questions. For example, the map will identify the key categories of societal impacts that have been researched and which types of ecological and societal impacts most often co-occur in the evidence. It will also reveal gaps or bias in the research, which may relate to geographical distribution, study type or category of impact or outcome.

The database will allow users to filter, search and sort the evidence and will contain the full coding for each study. Additionally, tables, charts and statistics will be available to summarise, group and visualise the evidence. Knowledge gaps and clusters in the research will be shown on an interactive evidence map using EPPI-Mapper software [24] thus allowing areas of further research to be identified and discussed. Study locations will be shown on a GIS map with markers indicating location and scale category of each study.

The systematic map is primarily intended to generate preliminary knowledge to support the instigation of further synthetic research and improve its rigour by identifying potential biases in the existing literature, such as geographical bias. Knowledge clusters will be evaluated and discussed, forming themes for more detailed systematic reviews or quantitative analyses. For example, there could be knowledge clusters around specific societal impacts, particular species moving northward that have significant impacts on specific societies that could allow for a more detailed critical analysis of the research base.

The outputs of the map will be useful for researchers to identify key areas for further research and inter-disciplinary collaboration. They will also be useful for decision-makers and policy developers in identifying current societal impacts and potential future impacts to be mitigated. Based on evidence gaps and clusters identified in the map, the authors will make recommendations for priority research areas and reporting protocols to address the inter-linkages between borealisation and societal changes.

## Electronic supplementary material

Below is the link to the electronic supplementary material.

Additional File 1: Benchmark List for Search String Scoping

Additional File 2: Search String in Scopus and Web of Science Formats

Additional File 3: Coding Sheet

Additional File 4: ROSES form for Systematic Map Protocol

## Data Availability

No datasets were generated or analysed during the current study.

## Declarations

### Author positionality

Like Pirie et al. (2024) [29] the authors recognise that their positionality should be disclosed in order to enhance the transparency and credibility of the research process. They acknowledge how this is shaped by factors including their personal, political, social, and cultural backgrounds which may affect aspects of the research including the understanding and evaluation of the research subject. *Kate Baker* is a researcher from the UK who received her formal training up to Masters (MSc Sustainability) and through her work as a Research Assistant where she has worked on social science projects in relation to sustainability. Before embarking on a Master’s degree, Kate was a primary school teacher and Special Educational Needs Co-ordinator. *Helen Wheeler* is an Associate Professor who works at the interface of the ecological and social sciences. Her work centres on co-productive work with Indigenous communities in northern Canada and brokers work between ecological and social sciences. *Vera Hausner* is a Professor in Sustainability science and has a background in ecology and political science. She is an assessment lead of the societal implications of climate change report from the Arctic Council (2024–2027). She has worked transdisciplinary with Arctic residents in various projects in Alaska, Canada, Russia, Greenland, Iceland and Norway. She has an Indigenous Sámi and a Kven heritage. *Jennifer Ramsay* is an inter-disciplinary researcher whose work focuses on inter-linkages between ecological and climate change processes. She is currently researching the impacts of large herbivores on climate processes and also provides consultancy support to wildlife organisations on conservation grazing and greenhouse gas emissions.
