# Supplementary material for: What evidence exists on the interlinkages between ecological and societal impacts of borealisation of the arctic? A systematic map protocol
Source: Environ Evid. 2025 Aug 2;14:15. doi: 10.1186/s13750-025-00367-4 (PMC12317435; doi:10.1186/s13750-025-00367-4)
Supplement: Supplementary file 1 — Additional File 1: Benchmark List for Search String Scoping [file 13750_2025_367_MOESM1_ESM.docx]

**Benchmark List for Search String Scoping**

Key papers that were selected for search string scoping:

1. Cunsolo Willox, A., Harper, S.L., Ford, J.D., Landman, K., Houle, K., Edge, V.L., 2012. “From this place and of this place:” Climate change, sense of place, and health in Nunatsiavut, Canada. Soc. Sci. Med. 75, 538–547. <https://doi.org/10.1016/j.socscimed.2012.03.043> (1)
2. Descamps, S., Strøm, H., 2021. As the Arctic becomes boreal: ongoing shifts in a high-Arctic seabird community. Ecology 102, e03485. <https://doi.org/10.1002/ecy.3485> (2)
3. Ford, J.D., Pearce, T., Canosa, I.V., Harper, S., 2021. The rapidly changing Arctic and its societal implications. WIREs Clim. Change 12, e735. <https://doi.org/10.1002/wcc.735> (3)
4. Fossheim, M., Primicerio, R., Johannesen, E., Ingvaldsen, R.B., Aschan, M.M., Dolgov, A.V., 2015. Recent warming leads to a rapid borealization of fish communities in the Arctic. Nat. Clim. Change 5, 673–677. <https://doi.org/10.1038/nclimate2647> (4)
5. Huntington et al (2018) Huntington, H.P., Loring, P.A., Gannon, G., Gearheard, S.F., Gerlach, S.C., Hamilton, L.C., 2018. Staying in place during times of change in Arctic Alaska: the implications of attachment, alternatives, and buffering. Reg. Environ. Change 18, 489–499. <https://doi.org/10.1007/s10113-017-1221-6> (5)
6. Møller, E.F., Nielsen, T.G., 2020. Borealization of Arctic zooplankton—smaller and less fat zooplankton species in Disko Bay, Western Greenland. Limnol. Oceanogr. 65, 1175–1188. <https://doi.org/10.1002/lno.11380> (6)
7. Mueter, F.J., 2022. Arctic Fisheries in a Changing Climate, in: Finger, M., Rekvig, G. (Eds.), Global Arctic: An Introduction to the Multifaceted Dynamics of the Arctic. Springer International Publishing, Cham, pp. 279–295. <https://doi.org/10.1007/978-3-030-81253-9_14> (7)
8. Myers-Smith, I.H., Forbes, B.C. & Wilmking, M. et al., 2011. Shrub expansion in tundra ecosystems: dynamics, impacts and research priorities. Environ. Res. Lett. 6, 045509. <https://doi.org/10.1088/1748-9326/6/4/045509> (8)
9. Pecuchet, L., Blanchet, M.-A., Frainer, A., Husson, B., Jørgensen, L.L., Kortsch, S., Primicerio, R., 2020. Novel feeding interactions amplify the impact of species redistribution on an Arctic food web. Glob. Change Biol. 26, 4894–4906. <https://doi.org/10.1111/gcb.15196> (9)
10. Roland, C., Schmidt, J.H., Stehn, S.E., Hampton-Miller, C.J., Nicklen, E.F., 2021. Borealization and its discontents: drivers of regional variation in plant diversity across scales in interior Alaska. Ecosphere 12, e03485. <https://doi.org/10.1002/ecs2.3485> (10)
11. Speed, J.D.M., Chimal-Ballesteros, J.A., Martin, M.D., Barrio, I.C., Vuorinen, K.E.M., Soininen, E.M., 2021. Will borealization of Arctic tundra herbivore communities be driven by climate warming or vegetation change? Glob. Change Biol. 27, 6568–6577. <https://doi.org/10.1111/gcb.15910> (11)
12. Ksenofontov, S., Backhaus, N., & Schaepman-Strub, G. (2018). ‘There are new species’: indigenous knowledge of biodiversity change in Arctic Yakutia. *Polar Geography*, *42*(1), 34–57. <https://doi.org/10.1080/1088937X.2018.1547326> (12)
13. Jaakkola, J.J.K., Juntunen, S. & Näkkäläjärvi, K. The Holistic Effects of Climate Change on the Culture, Well-Being, and Health of the Saami, the Only Indigenous People in the European Union. *Curr Envir Health Rpt* **5**, 401–417 (2018). <https://doi.org/10.1007/s40572-018-0211-2> (13)
14. Siwertsson, A., Lindström, U., Aune, M., Berg, E., Skarðhamar, J., Varpe, Ø., & Primicerio, R. (2024). Rapid climate change increases diversity and homogenizes composition of coastal fish at high latitudes. *Global Change Biology*, 30, e17273. <https://doi.org/10.1111/gcb.17273> (14)
15. A. Waits, A. Emelyanova, A. Oksanen, K. Abass, A. Rautio. 2018. Human infectious diseases and the changing climate in the Arctic. [Human infectious diseases and the changing climate in the Arctic - ScienceDirect](https://www.sciencedirect.com/science/article/pii/S0160412018312005) (15)
