# Supplementary material for: What evidence exists on the interlinkages between ecological and societal impacts of borealisation of the arctic? A systematic map protocol
Source: Environ Evid. 2025 Aug 2;14:15. doi: 10.1186/s13750-025-00367-4 (PMC12317435; doi:10.1186/s13750-025-00367-4)
Supplement: Supplementary file 2 — Additional File 2: Search String in Scopus and Web of Science Formats [file 13750_2025_367_MOESM2_ESM.docx]

**Additional File 2: Search Strings for Web of Science and Scopus**

Search String in Web of Science Format

(Arctic OR Canada OR Greenland OR "north*Finland" OR Iceland OR "north*Norway" OR "north*Sweden" OR Alaska* OR Russia OR Bering* OR Barents* OR "62° to 71°N" OR Polar OR Chukchi OR Siberia* OR "Laptev S*" OR "Kara S*" OR "White Sea" OR "Beaufort*" OR "Norwegian Sea" OR Labrador OR Baffin OR "Davis Strait" OR "Nares Strait" OR "Hudson Bay" OR "Hudson Strait" OR "Wandel Sea" OR "Lincoln S*" OR Lapland OR Lappland OR "MacKenzie River" OR "Ob River" OR "Lena River" OR "Yenise* River" OR Yukon OR "Kolyma River" OR “north slope” OR “Northwest Territories” OR Nunavut OR nunavik OR “nord-de-quebec” OR nome OR “wade hampton” OR bethel OR Dillingham OR Kamchatka OR Chukotka OR Taimyr* OR Taymyr OR Sakha OR Murmansk OR Yamal* OR Nenets* OR Troms* OR Nordland OR Finnmark* OR Norrland OR tundra OR "Attu station" OR "Pruhoe Bay" OR Adak OR Akutan OR Atka OR Fairbanks OR Kenai OR Kodiak OR Kotzebue OR McCharty OR Nikolski OR Seward OR Talkeetna OR Unalaska OR Wasilla OR "Aleutian Islands" OR "Nunivak Island" OR "St Lawrence Island" OR "St Matthew Island" OR "St. Lawrence Island" OR "St. Matthew Island" OR "Brooks Range" OR "Seward Peninsula" OR Yupik OR “Cambridge Bay" OR "Cape Dorset" OR "Clyde River" OR "Coral Harbour" OR "Gjoa Haven" OR "Goose bay" OR "Goose-bay" OR "Haines Junction" OR "Happy Valley" OR "Hay River" OR "Norman Wells" OR "North West River" OR "Rankin Inlet" OR "Repulse Bay" OR "Sawmill Bay" OR "Dawson city" OR Deline OR Igloolik OR Inuvik OR Iqaluit OR Ivujivik OR Kugluktuk OR Kuujjuaq OR Pangnirtung OR Salluit OR Tuktoyaktuk OR Ulukhaktok OR Whitehorse OR Wrigley OR Yellowknife OR "Amund Ringnes Island" OR "Axel Heiberg Island" OR "Banks Island" OR "Bathurst Island" OR "Bylot Island" OR "Devon Island" OR "Ellef Ringnes Island" OR "Ellesmere Island" OR "King William Island" OR "Prince Charles Island" OR "Prince of Wales Island" OR "Prince Patrick Island" OR "Queen Elizabeth Islands" OR "Somerset Island" OR "Southampton Island" OR "Victoria islands" OR "Mackenzie mountains" OR "North west territores" OR "Northwest territores" OR Inuvialuit OR Kitikmeot OR Kivalliq OR Nunatsiavut OR NunatuKavut OR Nunavik OR Nunavut OR Qikiqtaaluk OR Ungava OR "Faroe Islands" OR Faeroe OR Enare OR Ivalo OR Kemi OR Kittila OR Kuusamo OR Rovaniemi OR Torneå OR Ulea OR Uleåborg OR Inari OR "Torne river" OR "north magnetic pole" OR subarctic OR "Søndre Strømsfjord" OR Ilulissat OR Narssarssuaq OR Nuuk OR Sisimiut OR Umanak OR “kalaallit nunaat" OR qaanaaq OR Akureyri OR Isafjordur OR Reykjavik OR "Alta city" OR Bodoe OR Bodø OR Hammerfest OR Harstad OR Honningsvaag OR Honningsvag OR Karasjok OR Kautokeino OR Kirkenes OR Narvik OR Tomsoe OR “Jan Mayen" OR Lofoten OR Varanger OR "Alta river" OR "Pasvik river" OR "Tana River" OR Ivalojoki OR Karasjohka OR "Bristol Bay" OR "Resolute Bay" OR "Alpha ridge" OR "Amerasian bassin" OR "Amundsen Basin" OR "Amundsen Gulf" OR "Cumberland Sound"OR "Eurasian Basin" OR "Foxe Bassin" OR "Fram strait" OR "Gakkel Ridge" OR "Karskoje Sea" OR "Lancaster Sound” OR "Lomonosov Ridge" OR "Makarov bassin" OR "Mendeleev Ridge" OR "Mendeleev Rise" OR "Nansen Basin" OR "Northwest Passage" OR "Okhotsk Sea" OR "Siberian Sea" OR "Viscount Melville Sound" OR Beloye OR Storfjorden OR Anadyr OR Apatity OR Archangelsk OR Chatanga OR Chersky OR Dudinka OR Magadan OR Mirny OR Murmansk OR "Naryan-Mar" OR Nizhnevartovsk OR Norilsk OR Pevek OR Salekhard OR Severodvinsk OR "Tarko-Sale" OR Tiksi OR Urengoy OR Vorkuta OR Yakutsk OR “Franz Josef land" OR "Franz Josefs Land" OR "Novaya Zemlya" OR "Severnaja Zemlya" OR "Wrangel Island" OR Altai OR Anadyr OR Baikal OR "Chamar-Daban" OR Gydan OR Koryak OR Putoran OR Sayan OR "Tannu-Ola" OR Ural OR Verkhoyansk OR Yablonoi OR "Novosibirskije Ostrova" OR Yukaghir OR "Yamal-Nenets" OR Chukotka OR Evenkia OR "Jamalo-Nenetskij" OR Kamchatka OR Karelia OR "Khanty-Mansi" OR Kola OR Komi OR Koryakia OR Krasnoyarsk OR Sakha* OR Yakutia OR Magadan OR Murmansk OR Tunguska OR "Uvs Nuur" OR "Amur" OR "Anabar" OR "Angara" OR "Indigirka" OR "Irtysh" OR "Ob bay" OR "gulf of ob" OR Popigay OR Yana OR Longyearbyen OR "Ny Alesund" OR "Ny-Aalesund" OR "Nyalesund" OR "Ny-Alesund" OR "Ny-Ålesund" OR "Bear Island" OR Bjornoya OR Bjørnøya OR Spitsbergen OR Spitzbergen OR Svalbard OR Kongsfjord* OR Hornsund OR Abisko OR Aitik OR Arvidsjaur OR Gállok OR Gárasavvon OR Giron OR Gällivare OR Jokkmokk OR Kallak OR Karesuando OR Kiruna OR Korpilombolo OR Malmberget OR Muddus OR Pajala OR Ritsem OR Soppero OR "Stora sjöfallet" OR Svappavaara OR Vittangi OR Överkalix OR Akka OR Kebnekaise OR Norrbotten OR Padjelanta OR "Sarek" OR Tornedalen OR Tornedalian OR "Vindelfjällens naturreservat" OR "Lainio älv" OR Torneälven OR "Gwich’in" OR "Lower Tanana" OR "Tetlit Zheh" OR Ahtna OR Aklavik)

AND (borealisation OR borealization OR Atlantification OR pacification OR northward* OR "expan* taxa" OR "shifting taxa" OR poleward* OR "ice movement" OR "higher elevation" OR green* OR "climate change" OR "changing climate" OR "environmental change" OR extremes OR warming OR pulse* OR perturbation OR bloom* OR "coastal darkening" OR nitrification)

AND (population* OR species OR communit* OR organisms OR fish* OR game OR herbivore* OR bird* OR mammal* OR plant* OR tree* OR food OR wildlife OR predators OR environment* OR "eco-system" OR "ecoclimatic zone" OR "biome" OR invertebrates OR insects OR arthropods OR disease OR pathogen OR benthic OR pelagic OR reptiles)

AND (societ* OR socia* OR communit* OR human OR "place-based" OR place OR people OR fisher* OR hunt* or fishing OR forag* OR trap* OR harvest* OR herd* OR livelihood OR elder* OR youth OR men OR women OR health OR wellbeing OR well-being OR vulnerability OR resilience)

Search String in Scopus Format

( TITLE-ABS-KEY ( arctic OR canada OR greenland OR ( north*finland ) OR iceland OR ( north*norway ) OR ( north*sweden ) OR alaska* OR russia OR bering* OR barents* OR ( 62° AND to AND 71°n ) OR polar OR chukchi OR siberia* OR ( laptev AND s* ) OR ( kara AND s* ) OR ( white AND sea ) OR beaufort* OR ( norwegian AND sea ) OR labrador OR baffin OR ( davis AND strait ) OR ( nares AND strait ) OR ( hudson AND bay ) OR ( hudson AND strait ) OR ( wandel AND sea ) OR ( lincoln AND s* ) OR lapland OR lappland OR ( mackenzie AND river ) OR ( ob AND river ) OR ( lena AND river ) OR ( yenise* AND river ) OR yukon OR ( kolyma AND river ) OR ( north AND slope ) OR ( northwest AND territories ) OR nunavut OR nunavik OR ( nord-de-quebec ) OR nome OR ( wade AND hampton ) OR bethel OR dillingham OR kamchatka OR chukotka OR taimyr* OR taymyr OR sakha OR murmansk OR yamal* OR nenets* OR troms* OR nordland OR finnmark* OR norrland OR tundra OR ( attu AND station ) OR ( pruhoe AND bay ) OR adak OR akutan OR atka OR fairbanks OR kenai OR kodiak OR kotzebue OR mccharty OR nikolski OR seward OR talkeetna OR unalaska OR wasilla OR ( aleutian AND islands ) OR ( nunivak AND island ) OR ( st AND lawrence AND island ) OR ( st AND matthew AND island ) OR ( st. AND lawrence AND island ) OR ( st. AND matthew AND island ) OR ( brooks AND range ) OR ( seward AND peninsula ) OR yupik OR ( cambridge AND bay ) OR ( cape AND dorset ) OR ( clyde AND river ) OR ( coral AND harbour ) OR ( gjoa AND haven ) OR ( goose AND bay ) OR ( goose-bay ) OR ( haines AND junction ) OR ( happy AND valley ) OR ( hay AND river ) OR ( norman AND wells ) OR ( north AND west AND river ) OR ( rankin AND inlet ) OR ( repulse AND bay ) OR ( sawmill AND bay ) OR ( dawson AND city ) OR deline OR igloolik OR inuvik OR iqaluit OR ivujivik OR kugluktuk OR kuujjuaq OR pangnirtung OR salluit OR tuktoyaktuk OR ulukhaktok OR whitehorse OR wrigley OR yellowknife OR ( amund AND ringnes AND island ) OR ( axel AND heiberg AND island ) OR ( banks AND island ) OR ( bathurst AND island ) OR ( bylot AND island ) OR ( devon AND island ) OR ( ellef AND ringnes AND island ) OR ( ellesmere AND island ) OR ( king AND william AND island ) OR ( prince AND charles AND island ) OR ( prince AND of AND wales AND island ) OR ( prince AND patrick AND island ) OR ( queen AND elizabeth AND islands ) OR ( somerset AND island ) OR ( southampton AND island ) OR ( victoria AND islands ) OR ( mackenzie AND mountains ) OR ( northwest AND territores ) OR ( northwest AND territores ) OR inuvialuit OR kitikmeot OR kivalliq OR nunatsiavut OR nunatukavut OR nunavik OR nunavut OR qikiqtaaluk OR ungava OR ( faroe AND islands ) OR faeroe OR enare OR ivalo OR kemi OR kittila OR kuusamo OR rovaniemi OR torneå OR ulea OR uleåborg OR inari OR ( torne AND river ) OR ( north AND magnetic AND pole ) OR subarctic OR ( søndre AND strømsfjord ) OR ilulissat OR narssarssuaq OR nuuk OR sisimiut OR umanak OR ( kalaallit AND nunaat ) OR qaanaaq OR akureyri OR isafjordur OR reykjavik OR ( alta AND city ) OR bodoe OR bodø OR hammerfest OR harstad OR honningsvaag OR honningsvag OR karasjok OR kautokeino OR kirkenes OR narvik OR tomsoe OR ( jan AND mayen ) OR lofoten OR varanger OR ( alta AND river ) OR ( pasvik AND river ) OR ( tana AND river ) OR ivalojoki OR karasjohka OR ( bristol AND bay ) OR ( resolute AND bay ) OR ( alpha AND ridge ) OR ( amerasian AND bassin ) OR ( amundsen AND basin ) OR ( amundsen AND gulf ) OR ( cumberland AND sound ) OR ( eurasian AND basin ) OR ( foxe AND bassin ) OR ( fram AND strait ) OR ( gakkel AND ridge ) OR ( karskoje AND sea ) OR ( lancaster AND sound ) OR ( lomonosov AND ridge ) OR ( makarov AND bassin ) OR ( mendeleev AND ridge ) OR ( mendeleev AND rise ) OR ( nansen AND basin ) OR ( northwest AND passage ) OR ( okhotsk AND sea ) OR ( siberian AND sea ) OR ( viscount AND melville AND sound ) OR beloye OR storfjorden OR anadyr OR apatity OR archangelsk OR chatanga OR chersky OR dudinka OR magadan OR mirny OR murmansk OR ( naryan-mar ) OR nizhnevartovsk OR norilsk OR pevek OR salekhard OR severodvinsk OR ( tarko-sale ) OR tiksi OR urengoy OR vorkuta OR yakutsk OR ( franz AND josef AND land ) OR ( franz AND josefs AND land ) OR ( novaya AND zemlya ) OR ( severnaja AND zemlya ) OR ( wrangel AND island ) OR altai OR anadyr OR baikal OR ( chamar-daban ) OR gydan OR koryak OR putoran OR sayan OR ( tannu-ola ) OR ural OR verkhoyansk OR yablonoi OR ( novosibirskije AND ostrova ) OR yukaghir OR ( yamal-nenets ) OR chukotka OR evenkia OR ( jamalo-nenetskij ) OR kamchatka OR karelia OR ( khanty-mansi ) OR kola OR komi OR koryakia OR krasnoyarsk OR sakha* OR yakutia OR magadan OR murmansk OR tunguska OR ( uvs AND nuur ) OR amur OR anabar OR angara OR indigirka OR irtysh OR ( ob AND bay ) OR ( gulf AND of AND ob ) OR popigay OR yana OR longyearbyen OR ( ny AND alesund ) OR ( ny-aalesund ) OR nyalesund OR ( ny-alesund ) OR ( ny-ålesund ) OR ( bear AND island ) OR bjornoya OR bjørnøya OR spitsbergen OR spitzbergen OR svalbard OR kongsfjord* OR hornsund OR abisko OR aitik OR arvidsjaur OR gállok OR gárasavvon OR giron OR gällivare OR jokkmokk OR kallak OR karesuando OR kiruna OR korpilombolo OR malmberget OR muddus OR pajala OR ritsem OR soppero OR ( stora AND sjöfallet ) OR svappavaara OR vittangi OR överkalix OR akka OR kebnekaise OR norrbotten OR padjelanta OR sarek OR tornedalen OR tornedalian OR ( vindelfjällens AND naturreservat ) OR ( lainio AND älv ) OR torneälven OR ( gwich’in ) OR ( lower AND tanana ) OR ( tetlit AND zheh ) OR ahtna OR aklavik )

AND TITLE-ABS-KEY ( borealisation OR borealization OR atlantification OR pacification OR northward* OR ( expan* AND taxa ) OR ( shifting AND taxa ) OR poleward* OR ( ice AND movement ) OR ( higher AND elevation ) OR green* OR ( climate AND change ) OR ( changing AND climate ) OR ( environmental AND change ) OR extremes OR warming OR pulse* OR perturbation OR bloom* OR ( coastal AND darkening ) OR nitrification ) AND TITLE-ABS-KEY ( population* OR species OR communit* OR organisms OR fish* OR game OR herbivore* OR bird* OR mammal* OR plant* OR tree* OR food OR wildlife OR predators OR environment* OR ( eco-system ) OR ( ecoclimatic AND zone ) OR biome OR invertebrates OR insects OR arthropods OR disease OR pathogen OR benthic OR pelagic OR reptiles ) AND TITLE-ABS-KEY ( societ* OR socia* OR communit* OR human OR place-based OR place OR people OR fisher* OR hunt* OR fishing OR forag* OR trap* OR harvest* OR herd* OR livelihood OR elder* OR youth OR men OR women OR health OR wellbeing OR well-being OR vulnerability OR resilience ) )
