# Supplementary material for: What evidence exists on the interlinkages between ecological and societal impacts of borealisation of the arctic? A systematic map protocol
Source: Environ Evid. 2025 Aug 2;14:15. doi: 10.1186/s13750-025-00367-4 (PMC12317435; doi:10.1186/s13750-025-00367-4)
Supplement: Supplementary file 4 — Additional File 4: ROSES form for Systematic Map Protocol [file 13750_2025_367_MOESM4_ESM.pdf]

# Additional File 4: ROSES Reporting Standards for Systematic Map Protocols

Haddaway NR, Macura B, Whaley P, and Pullin AS. 2017. ROSES for Systematic Map Protocols. Version 1.0. DOI: 10.6084/m9.figshare.5897284.

| number | Section / sub-section | Topic | Description | Further explanation | Checklist/Meta-data | Author response | Comments |
|---|---|---|---|---|---|---|---|
| 1 | Title | Title | indicate if it is an update/amendment: e.g. "A systematic map update | question. | Meta-data | What evidence exists on the interlinkages between ecological and social impacts of | |
| 2 | Type of review | Type of review | map update, systematic map amendment | updates [2] | Meta-data | Systematic Map | |
| 3 | Authors contacts | Authors contacts | authors must be provided. | | Checklist | Yes | |
| 4 | Abstract | Structured summary | Background, the context and purpose of the review, including the | | Checklist | Yes | |
| 5 | Background | Background | known. Protocol must indicate why this study was necessary and what it | links the intervention or exposure to the outcome. | Checklist | Yes | Conceptual Map |
| 6 | Stakeholder engagement | Stakeholder engagement | (e.g. in the formulation of the question) must be described and | | Checklist | Yes | |
| 7 | Objective of the review | Objective | applicable). | questions are usually linked to sources of heterogeneity (effect | Checklist | Yes | |
| 8 | | components | intervention(s)/exposure(s), comparator(s), and outcome(s). | For other question types see [4,5] | Meta-data | Yes | |
| | Methods | | | | | Yes | |
| 9 | Searches | Search strategy | | Details regarding search strategy testing should be provided. | Checklist | Yes | |
| 10 | | Search string | the string is formatted (e.g. Web of Science format) | | Meta-data | Yes | |
| 11 | | databases | List languages to be used in bibliographic database searches. | | Meta-data | English | |
| 12 | | Languages – grey literature | based search engines. | | Meta-data | English and translated websites | |
| 13 | | Bibliographic databases | Provide the number of bibliographic databases to be searched. | | Meta-data | Five | |
| 14 | | Web – based search engines | Provide the number of web – based search engines to be searched. | | Meta-data | One - Google Scholar | |
| 15 | | Organisational websites | Provide the number of organisational websites to be searched. | | Meta-data | Seven | |
| 16 | | comprehensiveness of the search | strategy was assessed (i.e. list of benchmark articles). | | Checklist | Yes | |
| 17 | | Search update | review. | performed more than two years prior to review completion. | Checklist | Yes | |
| 18 | inclusion criteria | Screening strategy | relevance/eligibility. | | Checklist | Yes | |
| 19 | | Consistency checking | including the levels at which consistency checking will be undertaken | | Checklist | Yes | |
| 20 | | Inclusion criteria | articles/studies. These must be broken down into the question key | | Checklist | Yes | |
| 21 | | Reasons for exclusion | reasons for exclusion. | | Checklist | Yes | |
| 22 | Critical appraisal | Critical appraisal strategy | validity (including assessment of individual studies and the evidence | Optional | Checklist | n/a | |
| 23 | | synthesis | synthesis. | Optional | Checklist | n/a | |
| 24 | | Consistency checking | tested. | Optional | Checklist | n/a | |
| 25 | Data extraction | strategy | (potentially providing forms/data sheets (ideally piloted), list if | | Checklist | Yes | |
| 26 | presentation | Narrative synthesis strategy | base in the form of descriptive statistics, tables (including SM database) | of their findings) must be avoided. May include a summary of the | Checklist | Yes | |
| 27 | | identification strategy | knowledge gaps (unrepresented or underrepresented subtopics that | | Checklist | Yes | |
| 28 | | independence | articles to be considered within the review) in decisions regarding | review should be prevented from unduly influencing inclusion | Checklist | Yes | |
| 29 | Declarations | Competing interests | review authors may have. | | Checklist | Yes | |

**References**

[1] James, K.L., Randall, N.P. and Haddaway, N.R., 2016. A methodology for systematic mapping in environmental sciences. Environmental Evidence, 5(1), p.7.

[2] Bayliss, H.R., Haddaway, N.R., Eales, J., Frampton, G.K. and James, K.L., 2016. Updating and amending systematic reviews and systematic maps in environmental management. Environmental Evidence, 5(1), p.20.

[3] Haddaway, N.R., Kohl, C., da Silva, N.R., Schiemann, J., Spök, A., Stewart, R., Sweet, J.B. and Wilhelm, R., 2017. A framework for stakeholder engagement during systematic reviews and maps in environmental management. *Environmental Evidence* , *6* (1), p.11.

[4] Collaboration for Environmental Evidence. 2018. Guidelines and Standards for Evidence synthesis in Environmental Management. Version 5.0. www.environmentalevidence.org/information-for-authors.

[5] Leeds Institute of Health Sciences. https://medhealth.leeds.ac.uk/info/639/information_specialists/1500/search_concept_tools. Accessed 12/11/2017.
